# Comparing traditional, immersive simulation with Rapid Cycle Deliberate Practice in postgraduate year 2 anesthesiology residents

**DOI:** 10.1186/s41077-021-00174-0

**Published:** 2021-05-26

**Authors:** Erin E. Blanchard, Lee Ann Riesenberg, Lisa B. Bergman, Michelle R. Brown, Emma C. O’Hagan, Shivani J. Patel, Tekuila R. Carter

**Affiliations:** 1 Department of Anesthesiology and Perioperative Medicine, University of Alabama at Birmingham, 625 19th Street South, QT 334, Birmingham, AL 35249-5980 USA; 2 UAB Clinical Simulation/Office of Interprofessional Simulation for Innovative Clinical Practice, University of Alabama at Birmingham, Birmingham, AL 35249-5980 USA; 3 Department of Health Services Administration, University of Alabama at Birmingham, Birmingham, AL 35249-5980 USA; 4 University of Alabama at Birmingham, Birmingham, AL 35249-5980 USA

**Keywords:** Rapid cycle deliberate practice, Simulation, Medical education, Anesthesiology residents

## Abstract

**Background:**

Rapid Cycle Deliberate Practice (RCDP) is an increasingly popular simulation technique that allows learners to achieve mastery of skills through repetition, feedback, and increasing difficulty. This manuscript describes the implementation and assessment of RCDP in an anesthesia residency curriculum.

**Methods:**

Researchers describe the comparison of RCDP with traditional instructional methods for anesthesiology residents' application of Emergency Cardiovascular Care (ECC) and communication principles in a simulated environment. Residents (n = 21) were randomly assigned to either Traditional or RCDP education groups, with each resident attending 2 days of bootcamp. On their first day, the Traditional group received a lecture, then participated in a group, immersive simulation with reflective debriefing. The RCDP group received education through an RCDP simulation session. On their second bootcamp day, all participants individually engaged in an immersive simulation, then completed the “Satisfaction and Self-Confidence in Learning” survey. Application of ECC and communication principles during the simulation was scored by a blinded reviewer through video review. Participants ended the bootcamp by ranking the experiences they found most valuable.

**Results:**

No significant differences were found in the different group members’ individual performances during the immersive simulation, nor in the experiences they deemed most valuable. However, the Traditional education group reported higher levels of satisfaction and self-confidence in learning in 5 areas (p = 0.004–0.04).

**Conclusions:**

Regardless of RCDP or Traditional education grouping, anesthesia residents demonstrated no difference in ECC skill level or perceived value of interventions. However, members of the Traditional education group reported higher levels of satisfaction and self-confidence in numerous areas. Additional RCDP opportunities in the anesthesia residency program should be considered prior to excluding it as an educational method in our program.

**Supplementary Information:**

The online version contains supplementary material available at 10.1186/s41077-021-00174-0.

## Background

Simulation in the aviation industry is well established, and in the areas of music and sport, Rapid Cycle Deliberate Practice (RCDP) is a matter of course for both individual and a team or group [1–3]. Simulation-based education has become standard practice in medical education. RCDP in medical education was first described by Hunt et al. in 2014 [4]. RCDP combines the principles of deliberate practice, directive feedback, and mastery learning while prioritizing the opportunity for the learner to repeat tasks the “right way” after corrective feedback [5]. During a simulated scenario, the instructor pauses the learner’s action when errors occur to provide directive, customized, evidence-based feedback. The scenario is reset to the point where the learners have the opportunity to perform in the manner prescribed by the instructor, sometimes repeatedly, until the desired action is performed. Feedback delivery includes identification of the error and prescription of corrective action through scripting, choreography of expected actions, or solution-oriented debriefing. Generally, RCDP is utilized as a training modality for content that is time sensitive, team based, and algorithmic.

Studies of RCDP as a training modality have used a variety of outcome measures, including qualitative learner satisfaction and learner confidence [5–7], established scoring tools for specific team behaviors [8], procedural assessment with validated assessment tools, and “time-to” skill performance [4, 5, 7]. Published results related to learner satisfaction generally support that the training modality is appreciated by learners and increases their confidence in their performance. Assessment of individual performance after RCDP has revealed inconsistent improvement when using measurement tools, but “time-to” measures after RCDP have shown significant improvement [4, 5, 7].

A recent systematic review of the literature focused on RCDP use in medical education identified only two published articles [4, 9, 10]. In addition, we identified 5 recently published medical education studies comparing RCDP to more traditional simulation [6–8, 11, 12]. All of these studies included medical education participants (medical students, residents, or fellows) and focused on resuscitation or sepsis. The participants were pediatric learners [6–8], first-year residents from 19 specialties [11], and medical students [12]. None of the identified studies focused on second-year anesthesiology residents.

Given these data on the use of RCDP in medical education and as part of ongoing program evaluation advocated by the Society for Simulation in Healthcare (SSH) and the International Association for Clinical Simulation and Learning (INACSL), we chose to assess the implementation of RCDP in our residency curriculum [13, 14]. The purpose of this paper is to describe the comparison of RCDP with our traditional instructional methods for anesthesiology residents’ application of Emergency Cardiovascular Care (ECC) and communication principles in a simulated environment. As a secondary focus, we sought to analyze how the order of the educational methods may influence learner satisfaction, self-confidence, and their perceived value related to educational activities presented with different training modalities.

## Methods

The study compares Traditional education with RCDP education on anesthesiology residents’ application of (ECC) and communication in a simulated environment. For the purposes of this paper and to create a shared mental model of the instruction, each of the learner groups received either “Traditional” or “RCDP” instruction which are broadly defined as follows: the “Traditional” instruction had a 90-min didactic (teaching) followed by a 60-min group, immersive simulation and debriefing (application), and survey completion (assessment); the “RCDP” instruction group completed the same elements with teaching and application occurring concurrently during a 2.5-h session as dictated by the iterative cycles used with RCDP. Additional curricular details are provided in the instructional design section.

An expert trained in curriculum development (LAR) and three experts in simulation education (EB, TC, LB) designed and implemented the curriculum. Resident ECC skills and communication were assessed by a trained simulation expert who was blinded to group allocation. Other measures were identified during a review of the literature and included previous experience with ECC [15], satisfaction/self-confidence [5], and learner’s experience ranking surveys [16].

### Study design and participants

After obtaining ethics approval from the University of Alabama at Birmingham (UAB) Institutional Review Board, members of the postgraduate year (PGY) 2 anesthesiology residency class (n = 21) were randomly assigned to one of two bootcamp groups: Traditional or RCDP education. All residents took part in the activities, occurring during July of 2019. A power calculation was not used to determine the sample size, as all of the 21 PGY2 residents were to be included in the study. Residents provided electronic consent for research, allowing analysis of their survey results and simulation recordings. All participants were previously certified in advanced cardiac life support. Each group participated in 2 days of bootcamp, with the 2 days occurring 2 weeks apart.

### Study setting

These activities took place at the University of Alabama at Birmingham’s Office of Interprofessional Simulation for Innovative Clinical Practice. This SSH-accredited simulation center is equipped with multiple simulation, debriefing, and control rooms, in addition to audiovisual capabilities. The PGY2 anesthesiology bootcamp is held annually, with all PGY2 anesthesiology residents receiving simulation and didactic content designed to help them successfully transition to anesthesiology clinical practice. Per the Accreditation Council for Graduate Medical Education [17], all PGY2 anesthesiology residents must have completed a minimum of 6 months of education that includes “experience in caring for inpatients in family medicine, internal medicine, neurology, obstetrics and gynecology, pediatrics, surgery or any of the surgical specialties, or any combination of these,” as well as 1–2 months’ experience in “critical care and emergency medicine.” Additionally, all PGY2 residents are required to have Basic and Advanced Cardiac Life Support certifications, with the latter including instruction and application of ECC elements.

### Instructional methods

**Fig. 1:**
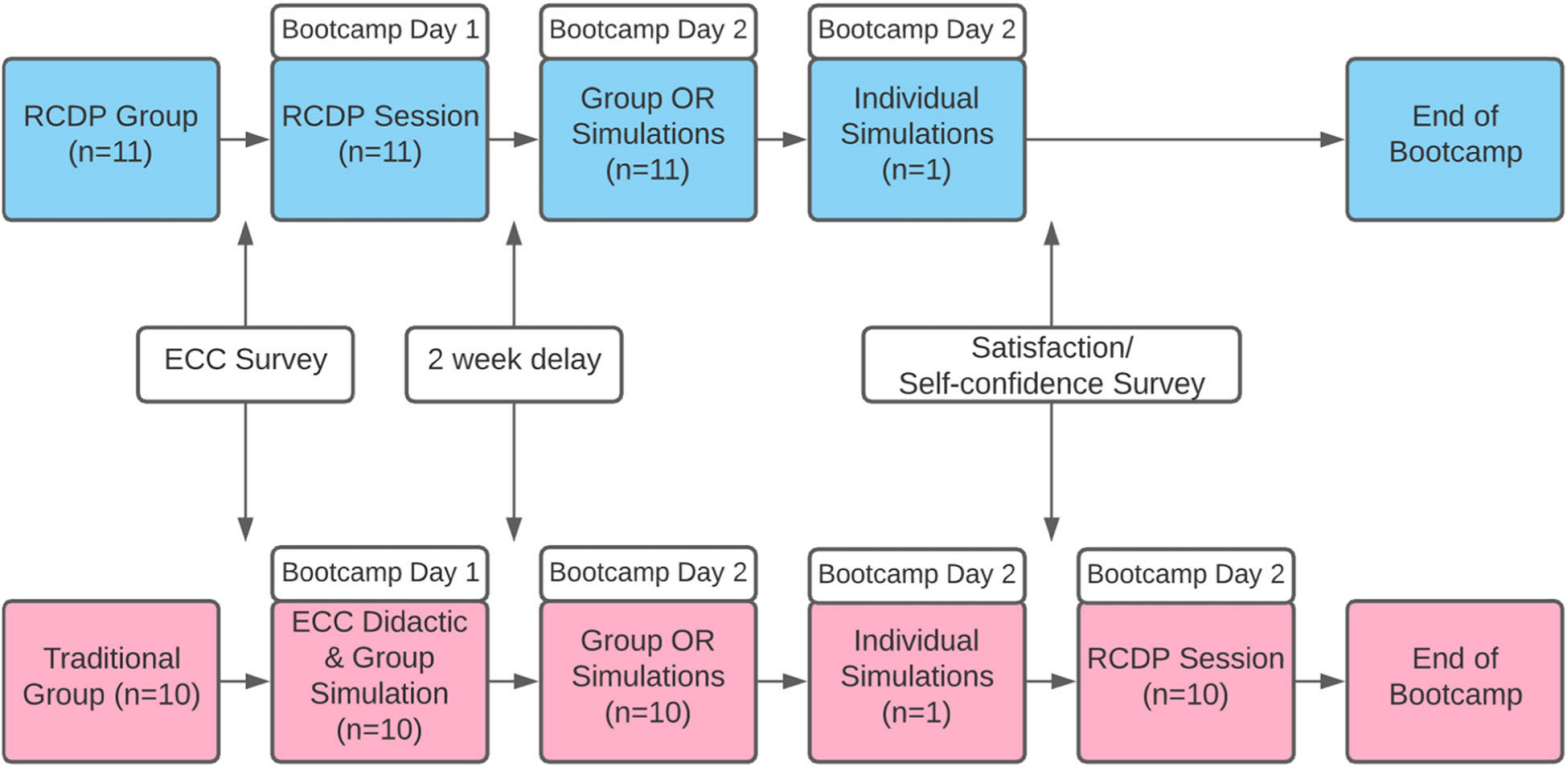
Bootcamp progression for RCDP and Traditional groups

Three educational elements were incorporated into each educational group: teaching, application, and assessment. The order of these elements differed as described hereafter. The study design is represented in Fig. 1.

### Bootcamp day 1

At the beginning of their first day of bootcamp, all participants completed the “Experience with ECC survey,” adapted from a previous article [11], evaluating baseline experience levels both in simulation and in clinical practice. The Traditional education group and the RCDP education group then received instruction as described below.

#### RCDP education group

On their first day of bootcamp, the *RCDP education* group (n = 11) received education through a 2.5-h RCDP simulation session (elements: teaching and application), where components of ECC, role assignment, closed-loop communication, and defibrillation operation were emphasized. Two facilitators extensively trained in RCDP and immersive simulation, as well as debriefing with good judgment, led the session. At the completion of the session, the participants completed the survey (element: assessment).

#### Traditional education group

On their first day of bootcamp, the *Traditional education* group (n = 10) received education through a 90-min lecture delivered by the same 2 previously mentioned facilitators (element: teaching). This lecture covered ECC interventions, closed-loop communication, assigning roles, and operating a defibrillator. Afterwards, residents took part in a group, immersive simulation (element: application), where they had the opportunity to utilize the skills covered in the preceding lecture on two patients requiring ECC. Following the simulation, a reflective debriefing session was conducted, exploring residents’ motivations and actions, and coaching on any identified gaps. The simulation and debriefing sessions lasted 60 min and were led by the same trained facilitators who conducted the RCDP simulation. At the completion of the session, the participants completed the survey (element: assessment).

### Bootcamp day 2

All participants, regardless of education group, engaged in an *individual*, *immersive simulation* where the patient required ECC interventions. This occurred after all subjects received education, either Traditional or RCDP, on their first day of bootcamp. A reflective debriefing, led by the same facilitators mentioned previously, followed the immersive simulation, after which residents were asked to complete the “Satisfaction and Self-Confidence in Learning” survey [18]. The individual, immersive simulation sessions were video recorded to enable retrospective review.

After completing the individual simulation, members of the Traditional education group received the same RCDP session that members of the RCDP group were exposed to on their first day of bootcamp, taught by the same instructors. At the end of their second day of bootcamp, members of both groups completed a ranking survey, adapted from a previously published article [16], asking them to classify the bootcamp activities in order of usefulness, with 7 being the most useful and 1 being the least useful.

### Simulation cases and performance assessment

The individual simulation scenario involved residents receiving a case stem stating that a patient was decompensating and needed their attention. Upon entering the room, residents were presented with a pulseless patient, showing a rhythm of ventricular fibrillation on the vital sign monitor. Residents were expected to initiate ACLS, delegate roles to trained embedded participants portraying nursing staff, and successfully defibrillate the patient. Upon defibrillation, the patient transitioned to unstable supraventricular tachycardia, requiring two rounds of synchronized cardioversion before converting into a normal sinus rhythm. When the patient converted to a normal sinus rhythm, or 10 min into the scenario, whichever came first, the scenario was ended. The participant then took part in a debriefing, followed by completion of the “Satisfaction and Self-Confidence in Learning” survey.

Using retrospective video review and blinding of participant identity and group, a trained simulation educator scored residents’ performances using a 24-item instrument, with 8 items adapted from the American Heart Association’s “Megacode Testing Checklist” [19]. This instrument, detailed in an additional file (see Additional file 1), was used to assess each learner’s performance related to defibrillation, synchronized cardioversion, Basic Life Support measures, and communication.

### Data management

All instruments were electronic and used the Research Electronic Data Capture (REDCap) system. Each participant was assigned a random participant code at the outset of the bootcamp. Each participant’s video tape was tagged with the participant’s codes by a different simulation expert (EB) than the person who ultimately reviewed them (LB). This allowed for the expert who analyzed each resident’s performance to be blinded to their name and group. The participant codes also allowed for anonymous comparison of survey results and performance metrics.

### Statistical analysis

Descriptive data were summarized using frequencies (percentages), with continuous data being analyzed using t-tests. Due to group size, Fisher’s test was utilized for analysis of dichotomous data. Each group’s mean rank order on the “Learners’ Experience Qualitative Tool” was analyzed using the Mann-Whitney test. All data were exported and analyzed using SPSS version 24, with two-sided p values <0.05 being statistically significant. Corrections were not made for multiple comparisons.

## Results

Twenty-one PGY2 anesthesiology residents participated in the study, with all completing each survey and taking part in the entire bootcamp. After randomization of the residents, 11 residents were assigned to the RCDP education group and 10 residents to the Traditional education group. No significant differences were found in the 2 groups’ baseline experience with ECC (Table 1).

**Table 1:** Comparison of two groups’ previous ECC experience

		RCDP (n = 11) group mean	Traditional (n = 10) group mean	p values
Did you have formal training in Code Management in Medical SchoolϮ		90.9%	70%	NS
Were you required to takeϮ				
	BLS-CPR in medical school	100%	100%	NS
	ACLS in medical school	40%	57.1%	NS
Were you exposed to a simulator in medical school?Ϯ		80%	85.7%	NS
	If yes, was it used to teach resuscitation skills? Ϯ	75%	83.3%	NS
Number of Real Codes attended*				
	During medical school	4.09	2.00	NS
	During residency	6.27	3.40	NS
Number of Codes attended*				
	On the floor	4.00	2.11	NS
	In the ICU	2.30	1.22	NS
	In the operating room	0	0	NS
	In the emergency department	.60	.44	NS
Number of times acting as Code Leader in a real code during residency*		.10	.11	NS
Have you personally defibrillated a patient?Ϯ		0%	0%	NS
Have you personally defibrillated a manikin?Ϯ		45.5%	60%	NS
	Number of times personally defibrillated a manikin*	1.60	1.00	NS
Have you personally synchronize cardioverted a manikin?Ϯ		27.3%	20%	NS
	Number of times personally synchronize cardioverted a manikin*	1.67	1.00	NS
Did you attend a mock code during your residency?Ϯ		27.3%	20%	NS
Have you personally synchronize cardioverted a patient?Ϯ		9.1%	10%	NS
	If yes, number of times personally synchronize cardioverted a patient*	1	1	NS

*t test demonstrates no difference (p > .05)

ϮFisher’s exact test demonstrates no difference (p > .05)

*NS* not significant

**Fig. 2:**
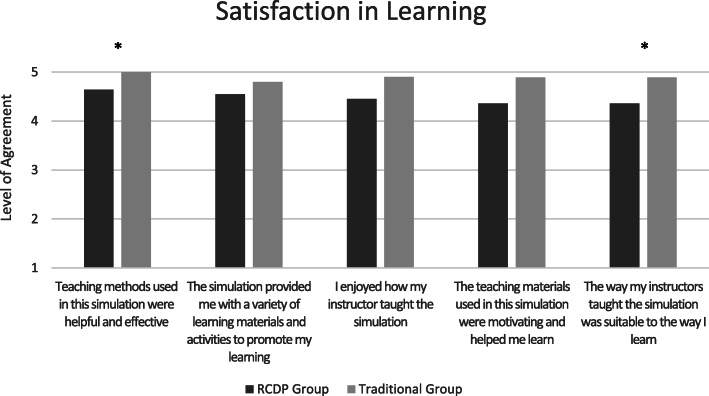
Bar charts illustrating the average scores obtained by Traditional and RCDP groups related to satisfaction on the Satisfaction and Self-Confidence in Learning questionnaire; learners were asked to rate how strongly they disagreed (1) or agreed (5) with each statement. *Significant at level of p = .04

**Fig. 3:**
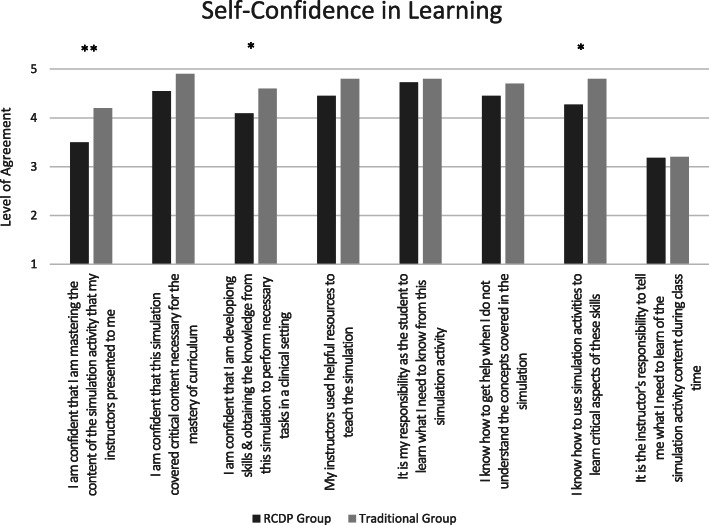
Bar charts illustrating the average scores obtained by Traditional and RCDP groups related to self-confidence on the Satisfaction and Self-Confidence in Learning questionnaire; learners were asked to rate how strongly they disagreed (1) or agreed (5) with each statement. *Significant at level of p = .04. **Significant at level of p = .004

**Fig. 4:**
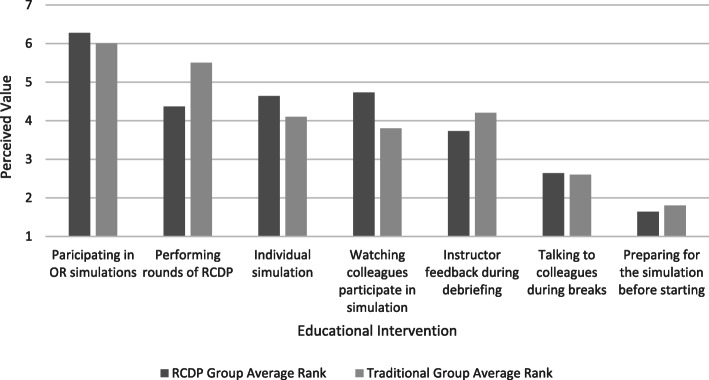
Learner’s Experience Qualitative Tool; learners asked to rank activities from which they learned the most (7) to which they learned the least (1)

When compared with the RCDP education group, the Traditional education group reported higher levels of satisfaction and self-confidence in learning in 5 areas (p = 0.004–0.04) (Figs. 2 and 3). No statistical differences were noted in participants’ evaluation of the usefulness of their experiences (Fig. 4) or their individual performances in the immersive simulation. However, members of the RCDP group were more likely to utilize the defibrillator in manual mode (p = .070), as opposed to Automated External Defibrillator (AED) mode, and members of the Traditional group defibrillated the patient an average of 65 s faster than the RCDP group (p = .081).

## Discussion

This study evaluated the use of Traditional versus RCDP education with PGY2 anesthesiology residents’ application of ECC and communication in a simulated environment. The goal was to use the outcomes to aid in curricular planning. According to The New World Kirkpatrick Model, understanding participant reactions to different educational approaches, assessing knowledge and skill acquisition, and appraising application during the simulation are all important parts of learner evaluation [20]. To assess these outcomes, residents completed surveys evaluating their satisfaction and self-confidence levels, along with perceptions of each activity’s value. A retrospective video review was conducted to assess each resident’s performance in a simulated clinical scenario involving the application of ECC content. Data showed that residents in the Traditional group had higher levels of satisfaction in the content and self-confidence in their skills than those in the RCDP group. No difference was found in which activities group members deemed most valuable, nor were there any differences in performance in a simulated setting.

Participants in the Traditional education group reported being more satisfied with their educational experience (Fig. 2) and self-confident in their skills in numerous areas (Fig. 3). This is consistent with a previous study where experienced learners preferred immersive simulation to RCDP [21]. Potential reasons for this difference could include an inherent skepticism in the RCDP group regarding a newly introduced educational approach. Although previously exposed to numerous immersive simulations, the bootcamp was the first time any of the residents were exposed to RCDP. Some learner groups have expressed frustration with their first RCDP interaction, followed by more positive perceptions in subsequent exposures [16]. As such, future research could involve exposing residents to RCDP earlier and more often before attempting to measure its effectiveness.

Additionally, the Traditional group received the RCDP session at the end of day 2, leaving the RCDP group to receive less education than the Traditional group. This may have contributed to the reduced satisfaction of the RCDP group. The RCDP group had the opportunity to apply ECC and communication skills in subsequent immersive simulations. However, these occurred after the conclusion of the study and would not be reflected in the results.

Members of the two groups performed similarly during the individual, immersive simulations on day 2 of bootcamp. Although not statistically significant, members of the RCDP group were more likely to use the defibrillator in manual mode (p = .070), as opposed to AED mode. This is likely due to the amount of time during the RCDP session that these learners operated the defibrillator in manual mode. While members of the Traditional group were shown how to operate the defibrillator in manual mode, they did not have the opportunity for deliberate practice with this skill. Interestingly, although there was a trend for learners in the Traditional group to utilize the defibrillator in AED mode, learners in this group were able to defibrillate the patient an average of 65 s faster than the RCDP group (p =.081). This could be due to either group’s generalized lack of experience, making those who left the defibrillator in AED mode quicker at treating shockable rhythms.

In addition, while we found 5 statistically significant between-group differences on the Satisfaction and Self-Confidence survey, these differences are relatively small. Given the small sample sizes in our study, future research should attempt to validate these results with a larger participant group. Although all members of the PGY2 anesthesiology class were included, increasing the number of participants could potentially yield different results and add value to future research. The granular experience of learners within a format like RCDP is likely still quite variable between contexts. This limits the generalizability of any findings and is a considerable limitation of this study.

There were no statistically significant differences regarding which activities the two groups found most valuable (Fig. 4). However, members of the Traditional group, which received RCDP after completion of their individual simulations, classified RCDP simulation as more beneficial than did their counterparts. There are several potential reasons for these findings. It is possible that doing the individual simulation prior to RCDP allowed the residents to self-identify any knowledge gaps or opportunities for improvement. The approach of preceding an RCDP session with an immersive simulation has been utilized with other populations and, in this case, potentially made members of the Traditional group more receptive to feedback and coaching [7, 11, 15]. Future research could include an initial immersive simulation for both groups to control for this possible variable.

Furthermore, participants in the bootcamp were familiar with the embedded person (EB) from previous educational interventions, potentially resulting in residents’ confusion when she would not offer aid in their individual simulations. In the future, using an embedded person with which the learners were not familiar may be beneficial.

Although we were unable to find a difference in ECC skill level or perceived value of interventions, there were several lessons learned. When studying educational modalities, previous experience and the amount of educational time should be the same for both groups. Also, the use of unfamiliar individuals during the study procedures may reduce confusion, and the use of a larger sample size will increase the possibility for generalization.

Additionally, future research could involve qualitative analysis of RCDP and traditional immersive simulation qualities, such as what components create the most value, result in skills improvement, and reduce cognitive load [16]. As suggested by others, further research is needed in additional subspecialties, evaluating retention of skills after RCDP training, and examination of how these skills translate into clinical practice [10, 16].

## Conclusion

Regardless of RCDP or Traditional education grouping, anesthesia residents demonstrated no difference in ECC skill level or perceived value of interventions. However, members of the Traditional education group reported higher levels of satisfaction and self-confidence in numerous areas. This might reflect that new educational methods, such as RCDP, may feel uncomfortable when first introduced; however, feeling less satisfied does not mean the education was less effective. As this is a single experience, additional RCDP opportunities in the UAB anesthesia residency program should be considered prior to excluding it as an educational method in our program. Future studies include investigating the potential dose effect of RCDP simulation on various learner experience levels and measuring performance and perceptions of learners experienced with RCDP compared with other simulation modalities.

## Supplementary Information

**Additional file 1.** Immersive Simulation Checklist. Description: Checklist utilized to evaluate learners’ performances in the individual, immersive simulation through retrospective video review.

## Data Availability

The datasets used and/or analyzed during the current study are available from the corresponding author on reasonable request.

## Abbreviations

ECC: Emergency Cardiovascular Care
PGY: Postgraduate year
RCDP: Rapid Cycle Deliberate Practice
REDCap: Research Electronic Data Capture
