# Supplementary material for: Comparing traditional, immersive simulation with Rapid Cycle Deliberate Practice in postgraduate year 2 anesthesiology residents
Source: Adv Simul (Lond). 2021 May 26;6:20. doi: 10.1186/s41077-021-00174-0 (PMC8157720; doi:10.1186/s41077-021-00174-0)
Supplement: Supplementary file 1 — Additional file 1. Immersive Simulation Checklist. Description: Checklist utilized to evaluate learners’ performances in the individual, immersive simulation through retrospective video review. [file 41077_2021_174_MOESM1_ESM.docx]

| **Additional File 1. Immersive Simulation Checklist** |
| --- |
|  |

- What was the length of time (in seconds) from when the curtain opens to the room until:
  - a pulse was checked: ___________
  - compressions were initiated: ___________
  - the learner placed pads on the patient: ­­­­­­­­­­­­­____________
  - the learner gave epi to the patient: ­­­­­­­­­­­­­____________
  - the learner defibrillated the patient: ­­­­­­­­­­­­­____________
- Indicate yes/no for the following actions:

The LEARNER….

- - Uses closed loop communication YES NO
  - Establishes him/herself as leader YES NO
  - Assigns team members roles YES NO
  - Ensures a backboard placed under the patient YES NO
  - Ensures high-quality CPR at all times YES NO

Ventricular Fibrillation (VF)/Defibrillation

- - Learner ensures:
    - Appropriate airway management YES NO
    - Immediately resumes CPR after shock YES NO
  - Learner personally:
    - Recognizes VF YES NO
    - Clears before ANALYZE and SHOCK YES NO
    - Defibrillates patient YES NO
      - If yes, manual mode used YES NO
    - Identifies ROSC YES NO

Cardioversion 1

- - Learner recognizes unstable tachycardia YES NO
  - Prior to 1^st^ cardioversion:
    1. Limb leads placed on the patient YES NO
    2. Defibrillator ‘sync’ button was pushed YES NO
    3. Defibrillator was charged to 50-100 Joules YES NO
    4. Learner pushed & held energy button until delivered YES NO
  - If answers to #1-4 above were all “YES”:
    - What was the length of time (in seconds) from when the learner defibrillated the patient until the learner delivered the first successful synchronized cardioversion (all “yes’s” for #1-4):
  - Learner recognizes unstable tachycardia after cardioversion YES NO

Cardioversion 2

- - Prior to 2^nd^ cardioversion:
    1. Limb leads on patient YES NO
    2. Defibrillator ‘sync’ button was pushed YES NO
    3. Defibrillator was charged to 100 Joules YES NO
    4. Learner pushed & held energy button until delivered YES NO
  - If answers to #5-8 above were all “YES”:
    - What was the length of time (in seconds) from the time the first cardioversion was administered until the second successful synchronized cardioversion (all “yes’s” for #5-8):
  - Learner determined patient no longer in unstable tachycardia YES NO
